# Hospital Annual Meetings

**Published:** 1919-03-08

**Authors:** 


### HOSPITAL ANNUAL MEETINGS.

### St. Mary's Hospital, Plaistow.

At the annual Court of Governors of St. Mary's Hospital for Women and Children, Plaistow, both in-patients and out-patients, it was reported, had increased in number during the past year, the daily average of beds occupied being 42.80, an increase of 2.20 as compared with 1917. Out-patients' attendances totalled 23,359, as against 21,712 a year previously. Income had amounted to £6,289, including £1,420 derived from legacies, and was £578 in excess of expenditure, which reached a total of £5,710. That figure was £344 more than in 1917, and £1,160 greater than in 1914. Now that the war is over the Committee of Management hopes to proceed with its schemes for providing a new nurses' home and a modern out-patient department. King Edward's Hospital Fund has promised grants to the extent of £3,500 for that purpose. In addition, contributions, amounting to £850, have been received by the Committee, and the Matron's Nurses' Home Fund has reached the total of £5,747.

### Queen Mary's Hospital, West Ham.

At the annual meeting of this hospital, presided over by Mr. C. E. Leo Lyle, M.P., it was stated that the number of in-patients cared for during 1918 was 1,937, while the attendances of out-patients totalled 131,743. During the war 1,765 sick and wounded soldiers have been treated in the wards, and more than 6,000 in the out-patient department.

The Committee of the hospital have under consideration the erection of a nurses' home, which it is estimated will cost £10,000, and the building of a maternity home, for which Mr. Charles Lyle has given £10,000. A new wing is to be added to the institution as a memorial to the men of West Ham who have been killed in the war. A sum of £60,000 is to be aimed at for that purpose.

### City of London Maternity Hospital.

The report for 1918, adopted at the annual meeting, states that the financial position of the institution still gives cause for anxiety, inasmuch as during the four years of war it has been necessary to increase the loan from the bankers to £7,300. Income last year amounted to 8,805, and was less than the expenditure by £1,055. Patients admitted numbered 1,325, a decrease of 39 as compared with 1917. In addition, 290 women were cared for in their own homes. Medical practitioners and students to the number of 36 attended the midwifery practice at the hospital during the year. Sheriff William R. Smith, M.D., said the institution's Baby Welfare Centre, which was being carried on so satisfactorily, deserved the greatest attention and support. Grants for Centres such as the hospital possessed must be available to a much larger extent than they had been in the past.

### Central London Ophthalmic Hospital.

At the annual meeting at the hospital, under the presidency of Mr. T. Brittin Archer, it was reported that 386 patients had been admitted to the institution during the past year, when 274 major and 927 minor operations were performed. Out-patients numbered 12,436, with 30,256 attendances. In addition 946 accident cases were attended to. Both in- and out-patients came not only from London but from all the Home Counties. Disabled sailors and soldiers suffering from eye diseases had been treated at the hospital by arrangement with the Ministry of Pensions. Expenditure for 1918 exceeded income by £196.

